# Is Shame Hallucinogenic?

**DOI:** 10.3389/fpsyg.2017.01310

**Published:** 2017-08-03

**Authors:** Simon McCarthy-Jones

**Affiliations:** Department of Psychiatry, Trinity College Dublin Dublin, Ireland

**Keywords:** affect, auditory hallucinations, dissociation, evolution, hypervigilance, psychosis, schizophrenia

## Abstract

Research into the causes of “hearing voices,” formally termed auditory verbal hallucinations (AVH), has primarily focused on cognitive mechanisms. A potentially causative role for emotion has been relatively neglected. This paper uses historical and contemporary case studies of AVH to tentatively generate the hypothesis that shame can be a causal factor in the onset of AVH. Other sources of support for the generation of this hypothesis are then sought. First, evidence is examined for a role of shame in the etiology of post-traumatic stress disorder, a condition that is characterized by phenomena related to AVH (intrusions and dissociation) and in which a substantial minority of sufferers report AVH. Second, the effect on AVH of a psychological therapy specifically designed to counteract shame (Compassion Focused Therapy) is noted. The hypothesis generation process is then expanded to propose mechanisms that could mediate a relation between shame and AVH. It is proposed that employing absorbed or avoidant strategies to deal with shame may lead to AVH through mediating mechanisms such as rumination, suppression, and dissociation. Evolutionary reasons for a relation between shame and AVH are also proposed, including that AVH may be an evolved mechanism to encourage self-protective behaviors in the wake of trauma. It is concluded that existing research supports the generation of this paper's hypothesis, which is now worthy of dedicated empirical testing.

## Introduction

No-one really knows why people hear voices in the absence of an appropriate external stimulus. Variously referred to as auditory verbal hallucinations (AVH), hearing voices, and voice-hearing (McCarthy-Jones, 2012), this experience has been the topic of extensive recent investigation. Whilst progress has been made in understanding the cognitive mechanisms involved in AVH (Waters et al., 2012), equivalent progress has not been made in uncovering the role of emotion.

There are three potential roles for emotions in AVH. They could be a cause (Slade, 1976; Romme et al., 2009), a maintenance factor (Morrison, 1998), and/or a consequence of AVH (Norman et al., 1998; Peters et al., 2012; Turner et al., 2013). Cognitive models of AVH (Chadwick and Birchwood, 1994; Peters et al., 2012) have predominantly examined how beliefs about voices determine the emotional consequences of the experience. As a result, the potential for emotions to play a causal role in the development of AVH has been relatively neglected. Nevertheless, there have always been those who have argued for such a relation, from Sigmund Freud to Marius Romme (Romme et al., 2009; McCarthy-Jones, 2017).

There are two key ways in which emotions could play a role in causing AVH. First, emotions could influence what the voices say. It has long been contended that the content of hallucinations can reflect emotions that precede them (Garety et al., 2001). Recently, Corstens and Longden (2013) found that the content of 94% of the voices heard by people diagnosed with schizophrenia could be related to earlier emotionally overwhelming events. The content of the voices and adverse events often shared common emotions, such as low self-worth, anger, shame, and guilt. Two caveats should be made to this finding. First, the relation between someone's emotions and what their voices say appears to be weaker in neurological disorders than psychiatric disorders. In a study of AVH in patients with brain lesions, Braun et al. (2003) found that what the voices said did not “correspond to any apparent emotional or moral obsession of the patient (as seems to occur in psychosis).” (p. 433). Second, even if emotions can influence what voices say in some people with diagnosed psychiatric disorders, it does not mean it is the case for all such people. For example, when Hardy et al. (2005) examined AVH in people with a history of trauma, they found that 43% had no links between the emotional themes of their trauma (humiliation, guilt, and threat) and the content of their voices. Furthermore, Daalman et al. (2012) found that trauma was associated with voice-hearing *per se*, and not just voice-hearing involving negative emotional content. This raises some concerns over the idea that emotions preceding voice-hearing are simplistically related to the content of ensuing voices.

A second way in which emotions could causally relate to AVH is by causing the onset of AVH themselves. Over two thousand years ago, Aristotle proposed that emotions could cause hallucinations by changing sensory perception (McCarthy-Jones, 2012). Today, emotion has been proposed to cause AVH through a number of mechanisms, including deficits in emotional prosody comprehension leading to voice identity misidentification (Alba-Ferrara et al., 2012; Tucker et al., 2013), negative emotions causing hypervigiliance for threat and leading to auditory false positives (Dodgson and Gordon, 2009), an inability to express (Escher et al., 2004) or tendency to suppress emotions (Badcock et al., 2011) leading to this affect coming to manifest in the form of voices, and, in a related vein, AVH as a defense mechanism against overwhelming emotions (Romme et al., 2009). The evidence for all these proposals remains limited.

These two ways in which emotions could play a causal role in AVH (shaping content, triggering onset) are potentially dissociable. Emotions could cause the content but not the onset of voices, or cause the onset but not the content of voices. The existence and nature of any causal relations between emotions and AVH hence remains to be better understood. As a small step toward this, the current paper will generate the hypothesis that a specific type of emotion may play a causal role in the onset of (some) AVH. When causal roles for emotion in AVH have previously been considered, the types of emotions explored have typically been limited to anxiety and depression (Tien and Eaton, 1992; Smith et al., 2006), although other emotions have also been pinpointed, such as sadness (Nayani and David, 1996; Escher and Romme, 2010). In contrast, this paper will put forward the idea that another form of affect may cause the development of voice-hearing; shame.

This hypothesis is consistent with the clinical experience of some individuals working with patients diagnosed with psychotic disorders (Dodgson and Gordon, 2009; G. Dodgson, personal communication) and has been previously suggested by both myself (McCarthy-Jones, 2012, 2017) and Breggin (2015). However, this paper will draw together evidence from a range of sources to offer the most detailed justification for the generation of this hypothesis to date.

## What is shame?

When we think of threats, we typically think of physical threats. However, threats can also take a social form, namely the endangering of our reputation and social standing. Social threats imperil our basic human need to belong and to be valued. They diminish what Gilbert (1989) has called our social attention holding power—which Gilbert has defined as our ability to “elicit positive attention and social rewards in the form of approval, praise, acceptance, respect, admiration, and desire” (Gilbert, 1997; p. 118). This makes such threats a fundamental part of our consciousness and a driver of our behavior. As George Orwell put it, “my whole life…was one long struggle not to be laughed at” (Orwell, as cited in Gilbert, 1997, p. 127).

Experiences of being “disgraced, devalued, demoted, dishonored, degraded, discredited, humiliated, ridiculed, shunned, ostracized, or scorned” (Gilbert, 1997, p. 113) are signalled to us through the emotion of shame; the feeling there is something flawed, bad or worthless about us (Gilbert, 1989). A “spoiled identity” (Goffman, 1986) can be a key cause of shame. As our ideals and standards are typically derived from other people, shame is also linked to negative social comparisons (Gilbert, 1989). Indeed, shame has been referred to as the “affect of inferiority” (Kaufman, 1989, p. 16), a phrasing that highlights its relational nature.

Taking an evolutionary view, Gilbert and McGuire (1989) have argued that shame can be adaptive. In this view, shame evolved from older mechanisms whose purpose was to regulate social rank and social behavior. Shame in humans is the equivalent of an animal submission display (head down, eyes averted), experienced to cause the person to undertake behaviors that de-escalate conflict and avoid further harm. It is part of a primitive system of social defense that evolved to protect against aggression (Gilbert and McGuire, 1989). Indeed, eye gaze avoidance is one of the most basic defensive behaviors, explaining why Euripides wrote that the eyes are the abode of shame. It is therefore unsurprising that shame is associated with a desire to hide (Tangney et al., 1996). The emotion of shame is hence a social threat detection system warning us that other people are viewing us negatively and may punish us (Turner et al., 2013). This belief may come to be internalized, leading us to believe that we are contemptible, wretched and worthless failures. In such internal shame, we become both the judge and the judged (Matos et al., 2013a).

One important aspect of shame that differentiates it from more basic emotions (Ekman, 1992), such as anger and fear, is that it is a self-conscious emotion, It involves an awareness of the self. It shares this property with other emotions such as embarrassment, pride, and guilt (Feiring, 2005). Whereas, pride occurs when standards are met, shame, guilt, and embarrassment stem from standards being violated (Feiring, 2005). Shame is closely related to guilt, but can be differentiated from it. In guilt, focus is typically on the person who was transgressed or the relationship that was affected, rather than the self (Tangney et al., 1996). Guilt pushes us out into the world, in an attempt to activate efforts to repair the damage by either reparative action or punishment (Tangney et al., 1996). In contrast, shame encourages withdrawal and retreat. In addition to considering how shame differs to other emotions, it should also be noted that it is also closely related to, and even constituted by, other emotions. For example, anxiety is central to the shame experience (Gilbert, 1989). In summary, shame can be viewed as an involuntary response to an experience of oneself as an unattractive social agent, an undesirable self, or a self one does not wish to be, which triggers a need to limit possible self-devaluation, self-damage, and loss of status, through escape or appeasement (Gilbert, 1989).

The content of AVH often involve shame. As far back as the third century, in Athanasius' (296–373) *Life of Anthony*, it was noted how hallucinated voices may “accuse and cast shame upon us” (Athanasius, 1994). This is still the case today (Connor and Birchwood, 2013; Corstens and Longden, 2013; Woods, 2017). Connor and Birchwood (2013) found that 35% of the voices heard by their sample of people diagnosed with schizophrenia had a specific theme of shaming. Corstens and Longden (2013) found that 60% of the voices heard by people diagnosed with schizophrenia embodied feelings of shame and guilt. Not only does the content of AVH often involve shame, but the form of AVH is also similar to that of shame. Both involve the perspective of another on the self. Shame is a social experience, as are many AVH (Bell, 2013). Whether, these parallels between shame and AVH in terms of form and content extend to them sharing the same function, is something that will be returned to later in the paper (see “*Shame, voice-hearing, and evolution*”). The next issue that will be addressed is whether there is any evidence to justify the generation of the hypothesis that shame can play a causal role in the development of AVH.

## Why would we suspect a causal relation between shame and AVH?

Both historical and contemporary case studies suggest that shaming AVH-content can be linked to earlier experiences of shame. In 1845 the French psychiatrist Esquirol (1845) reported a patient with voices that “accuse him…are continually repeating in his ear that he has betrayed his trust—that he is dishonored, and that he can do nothing better than destroy himself.” The roots of such shaming voices appear to have been, somewhat unsurprisingly, in events that led to shame. Esquirol's patient was formerly the head of a large German city whose inhabitants caused disorder by attacking the French army. The patient felt so ashamed of this that he cut his throat with a razor. He survived, but then voice-hearing began.

A case report from 1916, in the midst of the First World War also suggests shaming voices may be caused by experiences of shame (Rows, 1916). The patient was a 31-year-old private, admitted to hospital hearing voices. The voices were of his brother, elder sister, and brother in-law, telling him what to do and what not to do. It emerged that 5 years earlier he had slept with a prostitute. At first he was not disturbed by this, but later he thought he could “detect a strangeness in the behavior of his family, as if they knew of his misdeed.” He then began to hear voices, including those of his brothers and sisters, which appeared to come from a wall.

To take another historical example, Laing and Esterson (1964) describe the case of a young woman called Ruby. She had been admitted to hospital, hearing voices that calling her “slut,” “dirty” and “prostitute.” Interviews with Ruby and her family led to the discovery that 6 months before she was admitted to hospital, Ruby had fallen pregnant. When her family found out, they reacted in the following way; “while trying to pump hot soapy water into her uterus, told her…what a fool she was, what a slut she was” (p. 121). Indeed, the family also told the interviewers themselves “with vehemence and intensity, that she [Ruby] was a slut and no better than a prostitute” (p. 121).

We can also find links between shaming AVH-content and prior experiences of shame in contemporary case studies. For example, Dodgson and Gordon (2009) report the case of Michael. He was admitted to hospital hearing voices calling him a “nonce” (which is slang for pedophile) and mistakenly thinking people thought he was a pedophile. It emerged that when Michael was 15 he had masturbated to a variety of sexual fantasies, one of which involved his 8-year old sister. In his twenties, he became concerned that this meant he was a pedophile. He felt intense shame and became anxious about what the consequences of other people finding out about this would be. He feared someone else finding out and being publicly labeled as a pedophile, as local pedophiles were already being attacked. Michael became convinced that someone might know what he did, and started to become hypervigilant for any signs of this. He persistently scanned the noise outside his house to see if anybody was calling him a “nonce.” In time, he came to hear a voice calling him precisely this. This meant Michael became even more anxious and hypervigilant, which affected his sleep. He coped by using drugs. As he had not disclosed his past to others, but was nevertheless hearing a voice calling him a nonce, he started to believe that other people could read his mind. This increased his hypervigilance and anxiety even further. He coped by not going out and continually listening to background noises to see if he could hear the word “nonce.” This made him ever more sleep deprived and anxious, creating a vicious circle that led to his eventual hospital admission. This case study not only suggests a relation between shame and AVH, but also provides a mediating mechanism; hypervigilance, due to its relation with both shame (Budden, 2009) and AVH (Dodgson and Gordon, 2009). We will return to other potential mediators later.

The above examples suggest a potential role for shaming events in the onset of AVH. Whilst experiencing traumas, such as childhood maltreatment, do not necessarily lead to shame (Feiring et al., 2002), they have a strong potential to. In the case of child sexual abuse, for example, this is because highly salient standards of conduct have been violated by the perpetrator (Feiring, 2005) and perpetrators often encourage shame in order to keep the victim silent (Deblinger and Runyon, 2005). Indeed, shame is experienced in the wake of many traumas, particularly sexual ones (Koss, 2000; Scarce, 2001; Deblinger and Runyon, 2005; Ahrens, 2006; Karan et al., 2014). If shame commonly follows trauma, and shame plays a role in the etiology of AVH, we would hence expect to see AVH following traumas. Consistent with this, recent research has suggested that traumatic life-events may be causative of AVH (Read and Argyle, 1999; Read et al., 2003; Janssen et al., 2004; McCarthy-Jones, 2011; Bentall et al., 2012; Kelleher et al., 2013). Furthermore, shame is a major theme of many AVH heard in the context of a diagnosis of post-traumatic stress disorder (PTSD), where it can again be linked to precipitating events that people felt shame over. For example, Mueser and Butler (1987) give details of veterans (of Korea/Vietnam) who had PTSD and heard voices. Many heard voices related to enemy combatants that they had shot and killed, which typically told them to kill themselves. Another example is given by Bosson (2011), who describes the case of James. He was in his mid-50 s, and after Hurricane Katrina he had waited on his roof for 3 days to be rescued. As he waited, he heard others calling for help. Although, after he was rescued, he managed to also rescue others, there were many he could not save. After this, he came to hear voices that cried out, trying to get his attention, wanting to be saved.

Such observations allow the tentative generation of the hypothesis that shame plays an etiological role in the development of some AVH, potentially mediating the relation between trauma and AVH. This proposal does not envision shame to be either a sufficient or necessary cause of AVH. Not all experiences of shame lead to AVH, and not all cases of AVH can be traced back to experiences linked to shame. The novelty of this hypothesis means that it has not yet been empirically tested. In lieu of such tests, this paper will proceed by examining if there is any other indirect or suggestive evidence that would support the generation of this hypothesis.

## Intimations of an association: how research supports the generation of the hypothesis that shame can cause AVH

Intimations of an association between shame and AVH can be found in the PTSD research literature. AVH are found in many patients with PTSD, although they have been best studied in military veterans with PTSD. Here, it has been found that 20–65% of veterans with PTSD experience AVH (Holmes and Tinnin, 1995; Anketell et al., 2010; Brewin and Patel, 2010; see McCarthy-Jones, 2017, for a review). As a result of this and other related findings, it has been argued that AVH should be considered a characteristic symptom of PTSD (McCarthy-Jones and Longden, 2015). AVH also share a number of common characteristics with the core PTSD symptom dimension of intrusions (Crompton et al., 2017). There is hence reason to suspect that mechanisms involved in the development of PTSD may also give us insights into the causes of AVH. An empirical association between shame and measures of PTSD severity could offer some indirect support for generating the hypothesis that shame can cause the onset of AVH.

There is a well-documented relation between shame and both PTSD symptomatology in general and the PTSD symptom most closely related to AVH; intrusions. These relations have not only been documented in cross-sectional studies (Leskela et al., 2002), but also in prospective and experimental ones. For example, in a longitudinal study, Feiring and Taska (2005) found that levels of shame in survivors of child sexual abuse prospectively predicted levels of intrusive recollections. Dorahy et al. (2013) found that the experimental induction of shame led to increased levels of intrusions. In terms of more general PTSD symptomatology, Andrews et al. (2000) examined what factors predicted whether victims of violent crime developed PTSD symptoms. A month after the crime, it was found that shame (feeling they should have done more to stop the attack, thinking that they looked bad to others during it, or carrying bodily signs of the crime) and anger with others predicted the levels of PTSD symptomatology. Six months after the trauma, only shame surrounding their experience of crime remained a predictor of symptoms.

Levels of affect that correlate with levels of shame, such as guilt and self-blame (Averill et al., 2002), have also been found to encourage PTSD to develop (Feiring et al., 2002). For example, Cantón-Cortés et al. (2011) found that female students who had experienced child sexual abuse and who blamed themselves had more severe PTSD symptoms than those who did not blame themselves. Yet, it is notable that a cross-sectional study by Leskela et al. (2002) found that greater levels of shame-proneness, but not guilt-proneness, were associated with more severe PTSD symptoms in traumatized former prisoners of war. This hints at a potentially specific relation between shame and PTSD symptomatology, and by extension, AVH.

Should shame cause AVH, then it would be predicted that psychological therapies that reduce shame should have the effect of improving AVH. Although trauma-focussed cognitive behavioral therapy has been found to reduce levels of shame in traumatized individuals (Deblinger et al., 2006), a particularly notable therapy in this context is compassion-focused therapy (CFT; Gilbert, 2010). This has an explicit focus on shame. CFT builds on the idea that people with high levels of shame find it hard to be self-supporting or self-reassuring, in part because they have never learnt to be this way, due to a history of being shamed and criticized. By helping people to develop self-compassion and self-soothing, shame and other perceived threats can reduced. Research has already found that the greater capacity someone hearing voices has to self-reassure themselves, the less shameful content their voices have (Connor and Birchwood, 2013). Preliminary evidence has also found that CFT “had a major effect on voice-hearer's hostile voices, changing them into more reassuring, less persecutory and less malevolent voices” (Mayhew and Gilbert, 2008, p. 133). The histories of the participants in their study were notable in relation to the current hypothesis. One “had a sexual secret that he was ashamed of, that led him to worry about others discovering this secret and then punishing and rejecting him.” Another heard a voice that told him he was a pedophile. These cases echo those discussed in the context of hypervigilance hallucinations above, again suggesting a role for shame in AVH.

## Generating mechanisms for the hypothesized relation

The justification for generating the hypothesis that shame can cause AVH would be strengthened if plausible mediators of the relation between shame and AVH could be identified. One set of potential mediators may lie in the specific ways that individuals attempt to deal with shame and the event(s) that generated it. Whether constructive or absorbed/avoidant strategies (Simon et al., 2010) are used to deal with the event(s) associated with shame may influence the likelihood of AVH ensuing. A “constructive strategy” would be an effortful process by which someone creates a coherent account of the shame-related events and makes adaptive meanings of them (Simon et al., 2016). This is likely to require both engaging with and distancing from the shame-related events, in a flexible way, so they can be processed effectively, without causing cognitive or emotional overload (Simon et al., 2016). In contrast, absorbed and avoidant strategies involve the use of inflexible approaches for engagement with and distancing from shame-related events (Simon et al., 2010) and may encourage AVH. In an absorbed strategy this inflexibility would manifest in excessive attention to the shame-related events, whilst the inflexibility in an avoidant strategy would take the form of automatic disengagement from memories, cognitions, and emotions associated with the shame-related event (Simon et al., 2010, 2016).

One specific avoidant strategy that could mediate the shame-AVH relation is dissociation. Dissociation is a common way in which people may try to deal with shame (Dorahy and Clearwater, 2012). It can be used to regulate or even eliminate feelings of shame (Talbot et al., 2004) and may come to be automatically undertaken (Dorahy and Clearwater, 2012). Shame is positively associated with dissociation in trauma survivors (e.g., Talbot et al., 2004; Dorahy et al., 2013) and has been experimentally shown to increase levels of dissociation (Dorahy et al., 2017). Furthermore, dissociation may mediate the relation between shame and cognitive intrusions (Dorahy et al., 2017). Given that intrusive thoughts (Morrison and Baker, 2000; Jones and Fernyhough, 2006), intrusions from memory (Brébion et al., 2010; Waters et al., 2012), and dissociation (Longden et al., 2012; Varese et al., 2012; Pilton et al., 2015) have all been linked to AVH, it is plausible that a dissociative reaction to shame may lead to cognitive intrusions that come to be experienced as AVH. Notably, certain types of trauma, such as child sexual abuse, are particularly strongly associated with both dissociation (Talbot et al., 2004) and AVH (Bentall et al., 2012).

Another avoidant strategy that could mediate the shame-AVH relation is suppression; a conscious attempt to keep associated thoughts and emotions out of awareness. This can have the ironic effect of causing these to rebound with greater vigor unexpectedly into consciousness (Wegner, 1989). Shame is associated with thought suppression (e.g., Matos et al., 2013b) and thought suppression has been linked to hallucination-proneness (Jones and Fernyhough, 2006). It is also notable that voice-hearers diagnosed with schizophrenia who try to suppress their emotions more, tend to hear more frequent and louder voices (Badcock et al., 2011). Suppression could hence also mediate between shame and AVH. One reason for undertaking emotional suppression is that social support is not available to help you deal with your emotions. This suggests that a lack of social support could also mediate the relation between shame and voice-hearing. Although, no research speaks directly to this, a lack of social support has been found to predict whether PTSD will develop after a trauma (Brewin et al., 2000).

Absorbed strategies for dealing with shame could also mediate the shame-AVH relation. Shame is associated with increased levels of rumination (e.g., Orth et al., 2006) and rumination is both associated with hallucination-proneness (Jones and Fernyhough, 2008), and prospectively predicts PTSD severity (Michael et al., 2006). One reason an individual may come to ruminate on perceived shame could be due to being socially isolated. It has previously been argued that social isolation is associated with AVH by Hoffman (2007), who accounted for this using the concept of social deafferentation. However, isolation encouraging rumination could be another mechanism to explain why this relation pertains.

If future research does establish that specific regulatory strategies employed to deal with shame contributed to the development of distressing AVH, it may be asked how alternative ways of regulating this emotion could be encouraged. The recent literature on emotional regulatory flexibility (Bonanno and Burton, 2013) suggests that this should involve three considerations; how can situational cues that avoidance or absorption are likely to be the most effective regulatory strategy be avoided, how can the person's repertoire of methods for regulating shame be widened, and how can the person's capacity to monitor feedback about how effective their choice of shame regulation strategy has been be increased? What appears likely to be important here is to recognize that the person's choice of regulation strategy may have initially been adaptive for them, particularly when the threat was still present, but that “what might be considered effective coping at the outset of a stressful situation may be deemed ineffective later on” (Folkman and Moskowitz, 2004, p. 754).

## Shame, voice-hearing, and evolution

Postulating a relation between shame and AVH also allows a further, often unasked, question to be addressed. AVH are typically portrayed, and indeed often experienced, as being detrimental to an individual's fitness. So, why have they not been eliminated by natural selection? One family of answers to this question are that AVH are due to exaggerations of other successful adaptations, such as synaptic pruning (Hoffman and McGlashan, 1997) or threat detection (Dodgson and Gordon, 2009). In a similar vein, I recently built on Frith and Metzinger (2016) argument that self-consciousness evolved because it allowed us to experience shame, to note that if this were true then we should not be surprised that powerful experiences of shame are associated with compelling contents of consciousness such as voice-hearing (McCarthy-Jones, 2017).

An alternative possibility, given the significant number of AVH that are experienced as shaming (Connor and Birchwood, 2013; Corstens and Longden, 2013) is that AVH are not a *cost* of another process, but rather are experiences that were selected for because they share the same *adaptive function* as shame. Breggin (2015) has already noted the parallel between the effects of AVH and the function of shame. Yet Breggin focusses on shame as a negative emotion, and does not consider how the potentially adaptive aspect of shame stressed by Gilbert and McGuire (1989) may apply to shaming AVH. If shame can be adaptive because it encourages submissive, self-protective behaviors, then AVH could have evolved as they too encourage self-protective behaviors. But why would a person hearing voices need to undertake such behaviors? This may be due to the strong relation between trauma and AVH, noted earlier. The evolutionary reason for AVH could hence be to facilitate experiences of shame in traumatized people, in order to encourage self-protective behaviors to aid survival. In such an account, AVH would not be a symptom of psychopathology, but an evolved mechanism to protect traumatized people from further harm. This idea would fit with the idea, proposed by many members of the Hearing Voices Movement, that hostile voices are in fact misunderstood messengers whose actual purpose is to support and protect the hearer (see McCarthy-Jones, 2017).

There are, of course, a number of important caveats here. First, this hypothesis in no way endorses the shaming of traumatized individuals. This would be to commit the naturalistic fallacy. Society should be working to overcome the shame surrounding suffering traumas such as abuse, not perpetuating it. Indeed, support from family and friends is one of the key predictors of resilience in the face of trauma (Collishaw et al., 2007). This hypothesis merely seeks to explain why voice-hearing evolved. Secondly, it would need to be explained why shaming voice-hearing persists after the trauma itself ends. As Coifman and Bonanno (2010) note, because emotions are argued to have evolved to help solve specific problems in specific situations (Tooby and Cosmides, 1990), they can be dysfunctional when they occur beyond such conditions. It would hence make sense for shame-inducing voices to stop once the threat had passed. Yet beneficial coping strategies forged in the crucible of trauma can be maintained when the environment normalizes, even when such strategies are then maladaptive e.g., (Folkman and Moskowitz, 2004). Alternatively, it may be that perceptions of threat continue long after the trauma ends, leading to the persistence of the protective, withdrawal-inducing shame. Clearly, this is highly speculative proposal, yet it may offer a fruitful new way to think about voice-hearing.

## Conclusions

**Figure 1:**
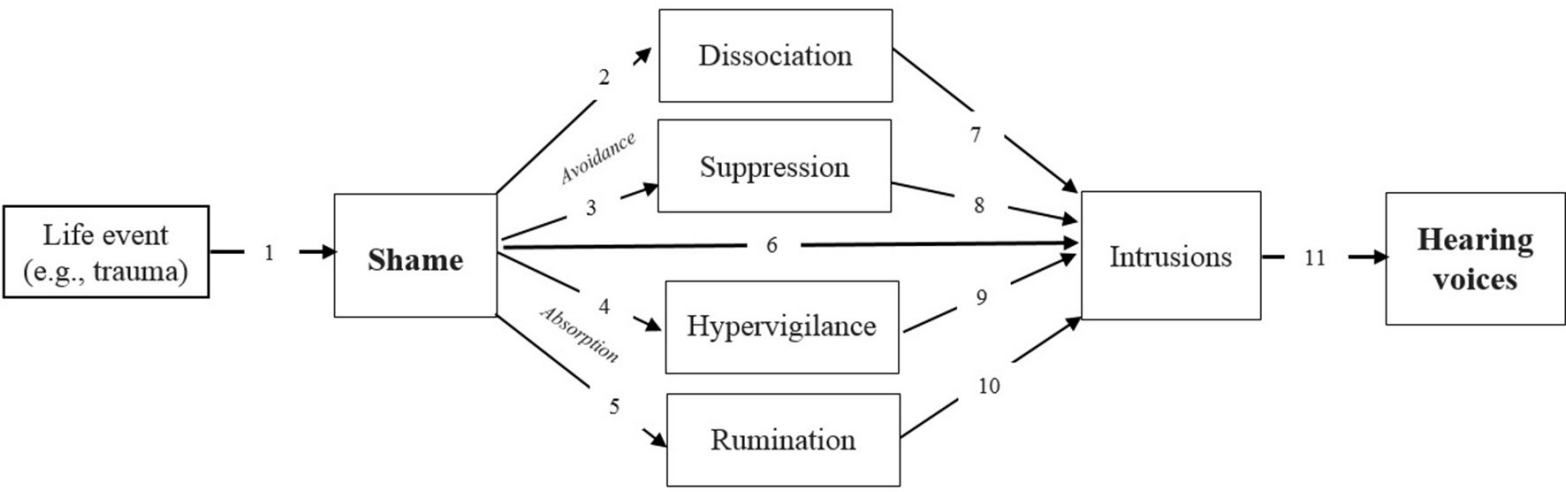
Constructing a hypothesized causal relation between shame and hearing voices. 1, Trauma → Shame (Koss, 2000; Scarce, 2001; Ahrens, 2006; Karan et al., 2014); 2, Shame → Dissociation (Talbot et al., 2004; Dorahy et al., 2013); 3, Shame → Suppression (Matos et al., 2013b); 4, Shame → Hypervigilance (Budden, 2009); 5, Shame → Rumination (Orth et al., 2006); 6, Shame → Intrusions (Feiring and Taska, 2005; Dorahy et al., 2017); 7, Dissociation → Intrusions (Dorahy et al., 2017); 8, Suppression → Intrusions (Wegner, 1989); 9, Hypervigilance → Intrusions (Watson and O'Hara, 2017); 10, Rumination → Intrusions (Jones and Fernyhough, 2008); 11, Intrusions → Hearing voices (Morrison and Baker, 2000; Jones and Fernyhough, 2006; Brébion et al., 2010; Waters et al., 2012).

The current paper has hypothesized a causal relation between shame and AVH, focussing on the potential for shame to play a role in the onset of AVH, not just their content. The research reviewed here that has contributed to the generation of this hypothesis is summarized in Figure 1. As no studies have directly investigated the potential causal role for shame in AVH, the evidence used for this hypothesis generation had to be drawn from the lower echelons of the hierarchy of evidence, such as case-studies and analogs from related phenomena such as PTSD. Nevertheless, given the range of plausible theoretical mechanisms that may mediate causal relation between shame and AVH, it seems to be a potentially fruitful hypothesis worthy of future testing.

Numerous specific hypotheses suitable for empirical testing can be extracted from this paper. A first set of hypotheses would simply focus on the relation between shame and the content of AVH. These would include the hypothesis that reducing shame in voice-hearers will reduce the shameful content of the AVH. A second series of hypotheses center on the proposition that shame plays a causal role in the onset of AVH after a trauma. These would include the hypothesis that shame mediates the relation between trauma and AVH. A third set of hypotheses relate to the specificity of the relation between shame and AVH. These include that shame, and not guilt, will mediate the relation between trauma and AVH. This hypothesis is based on evidence that shame, but not guilt, is associated with more severe PTSD symptomatology (Leskela et al., 2002), and that shame, but not guilt, mediates the treatment effects of psychological interventions for PTSD (Ginzburg et al., 2009). Other emotions that have been found to commonly precede day-to-day experiences of AVH include sadness (Nayani and David, 1996), and as shame is associated with sadness (Feiring, 2005) it is possible that many emotions currently known to be associated with voice-hearing may in fact be manifestations of underlying shame.

A key challenge facing future studies that attempt to test these hypotheses is how to measure shame. Not only is there currently no gold standard measure of shame, but the staple of psychological research, self-report questionnaires, may only accurately assess levels of shame in people with a high degree of objective self-awareness (Platt and Freyd, 2012). Shame may often present “in disguise,” being covertly signaled through the use of code-words (Rahm et al., 2006). As such future studies may wish to also employ non-verbal assessment measures (Bonanno et al., 2002), such as Feiring and Taska's (2005) Shame Posture Measure, which asks participants to rate to what degree drawings of shame postures represent how they currently feel. Furthermore, studies should likely use measures of both abuse-related shame and general measures of shame-proneness. Feiring and Taska (2005) found these measures are only weakly related, and it will need to be determined if both, only one, or neither of these types of shame are associated with AVH.

A causal role in AVH for an emotion such as shame would not negate a role for cognitions. For example, not only do direct emotion accounts of hallucinations propose that emotions may trigger cognitive changes, leading to AVH (Slade, 1976; Freeman and Garety, 2003), but such emotions are argued, in turn, to be preceded by cognitions (Stinson et al., 2010). Furthermore, not only is shame itself a complex mix of cognition and affect (Gilbert, 1997) but shame-proneness is likely to arise from negative schema about the self and others (Gilbert, 1989). There will hence be the need to understand the complex interplay between affect and cognition in any role for shame in causing AVH. Whether or not the specific hypotheses outlined in this paper are falsified by future empirical work, they appear to offer a fruitful new framework for conceptualizing and researching AVH in.

## Author contributions

The author confirms being the sole contributor of this work and approved it for publication.

### Conflict of interest statement

The author declares that the research was conducted in the absence of any commercial or financial relationships that could be construed as a potential conflict of interest.
